# Supplementary material for: Allele-specific gene expression can underlie altered transcript abundance in zebrafish mutants
Source: eLife. 2022 Feb 17;11:e72825. doi: 10.7554/eLife.72825 (PMC8884726; doi:10.7554/eLife.72825)
Supplement: Supplementary file 1. [file elife-72825-supp1.pdf]

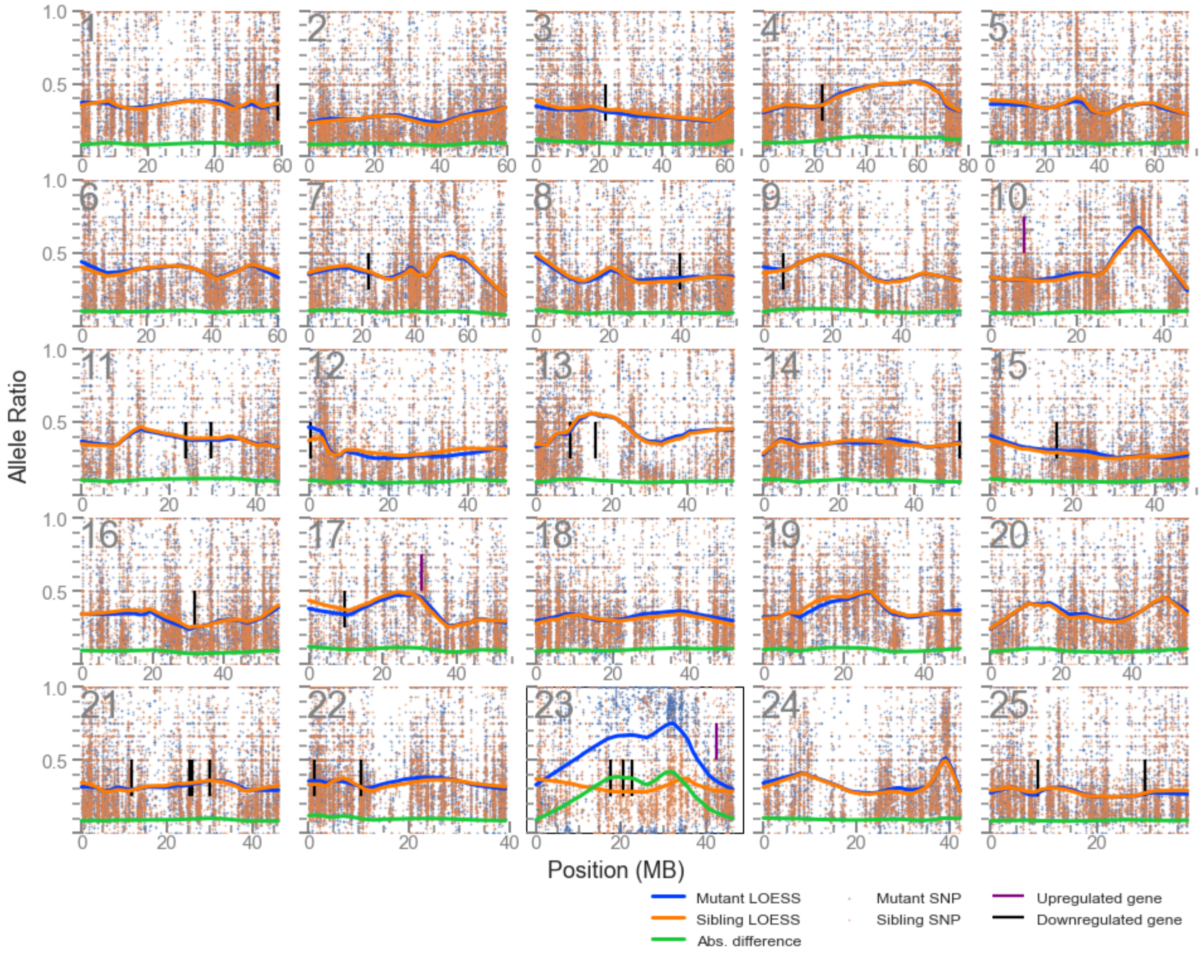

Allele u757 (A66) gene: unpublished (chr23, 22Mbp)

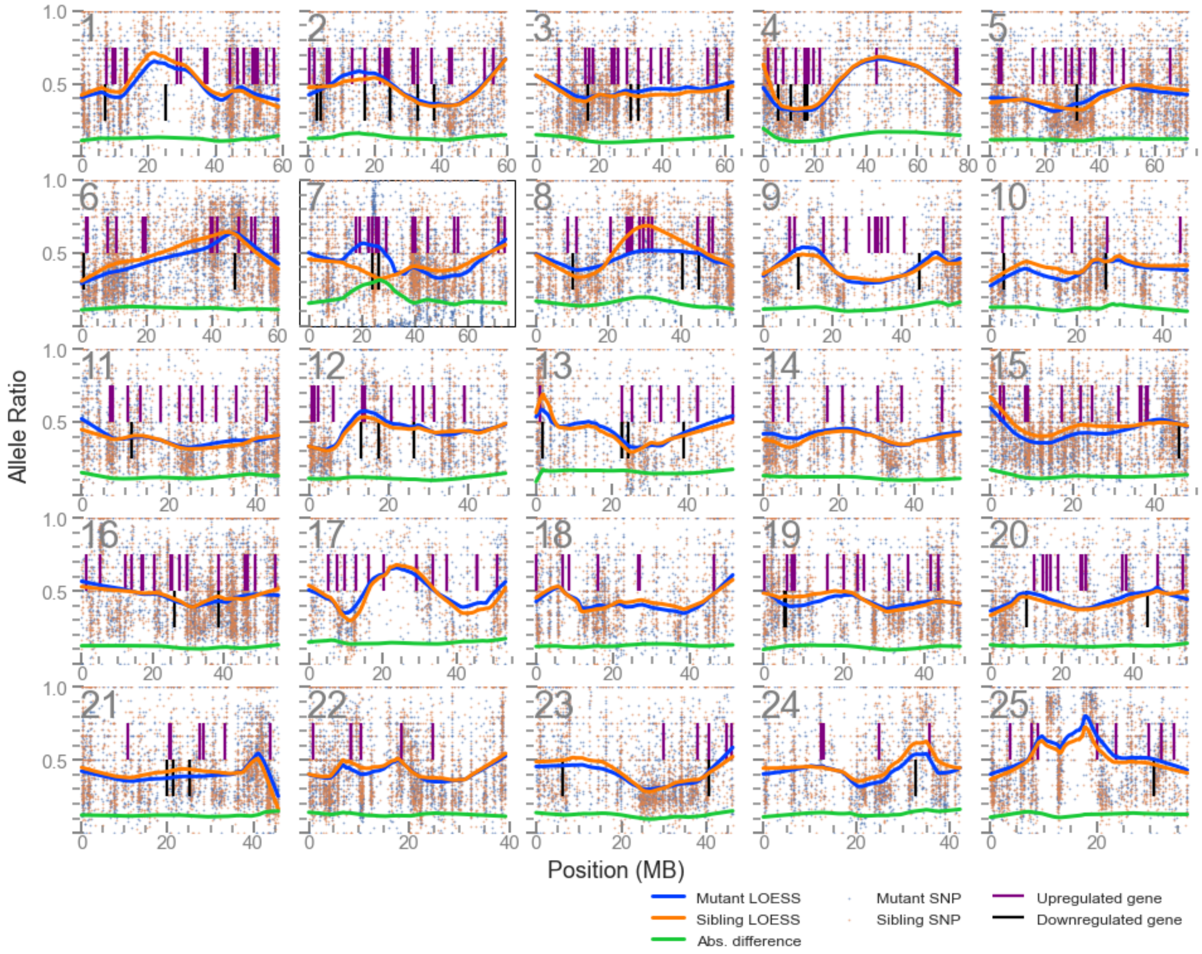

Allele sb55, gene: ache (chr 7, 26.0Mbp)

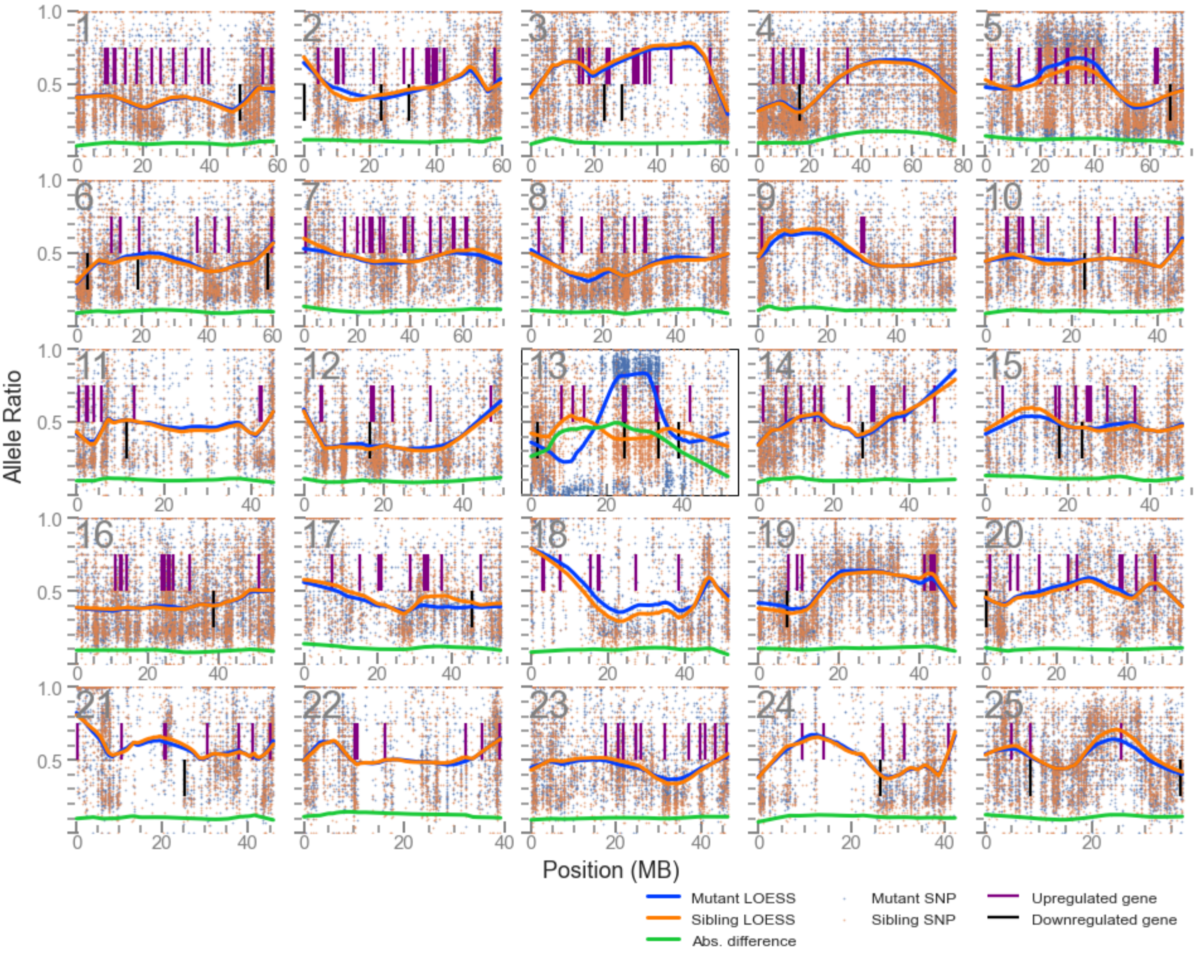

Allele u535 (B70), gene not known (chr13, ~25Mbp)

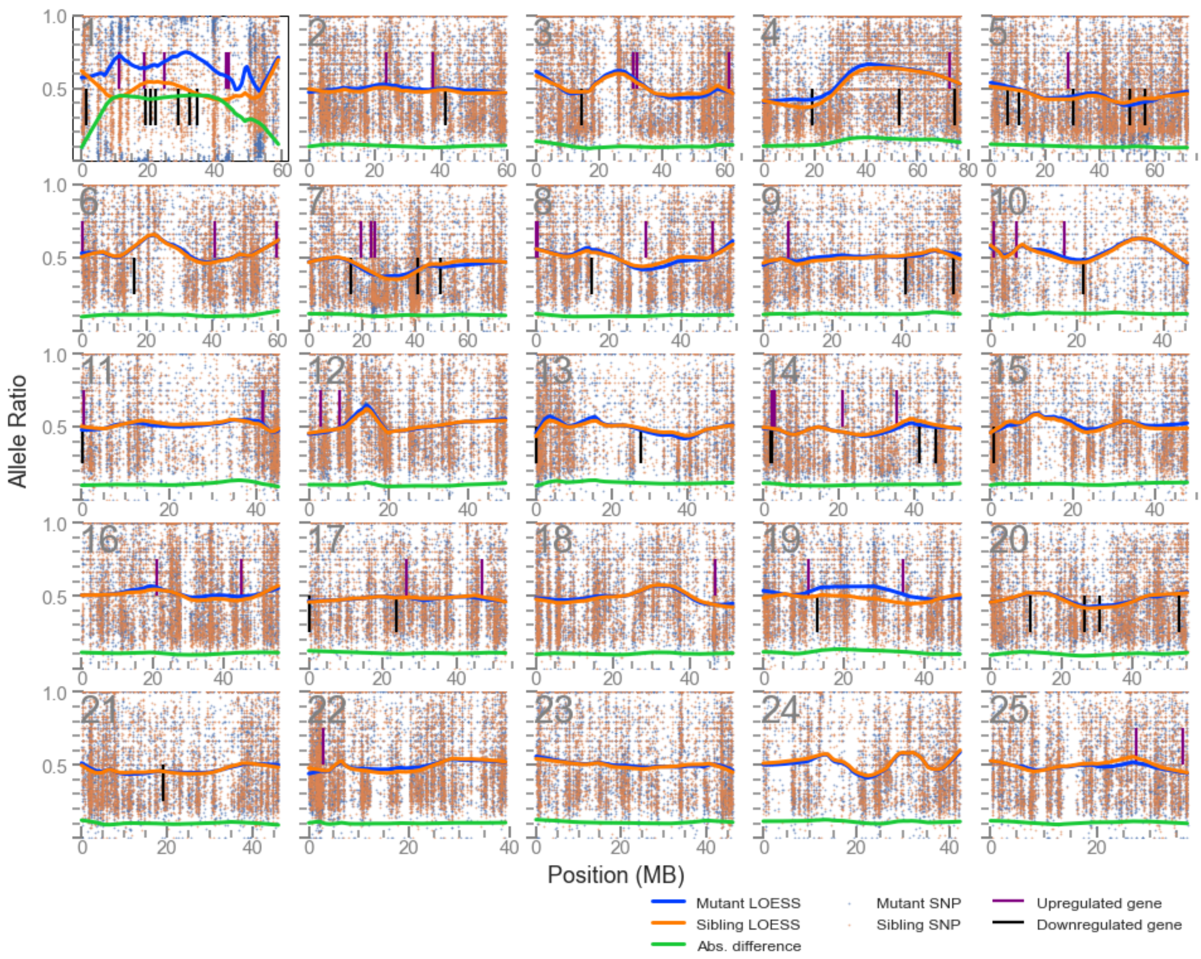

Allele u534 (C65), gene not known (chr1, ~25Mbp)

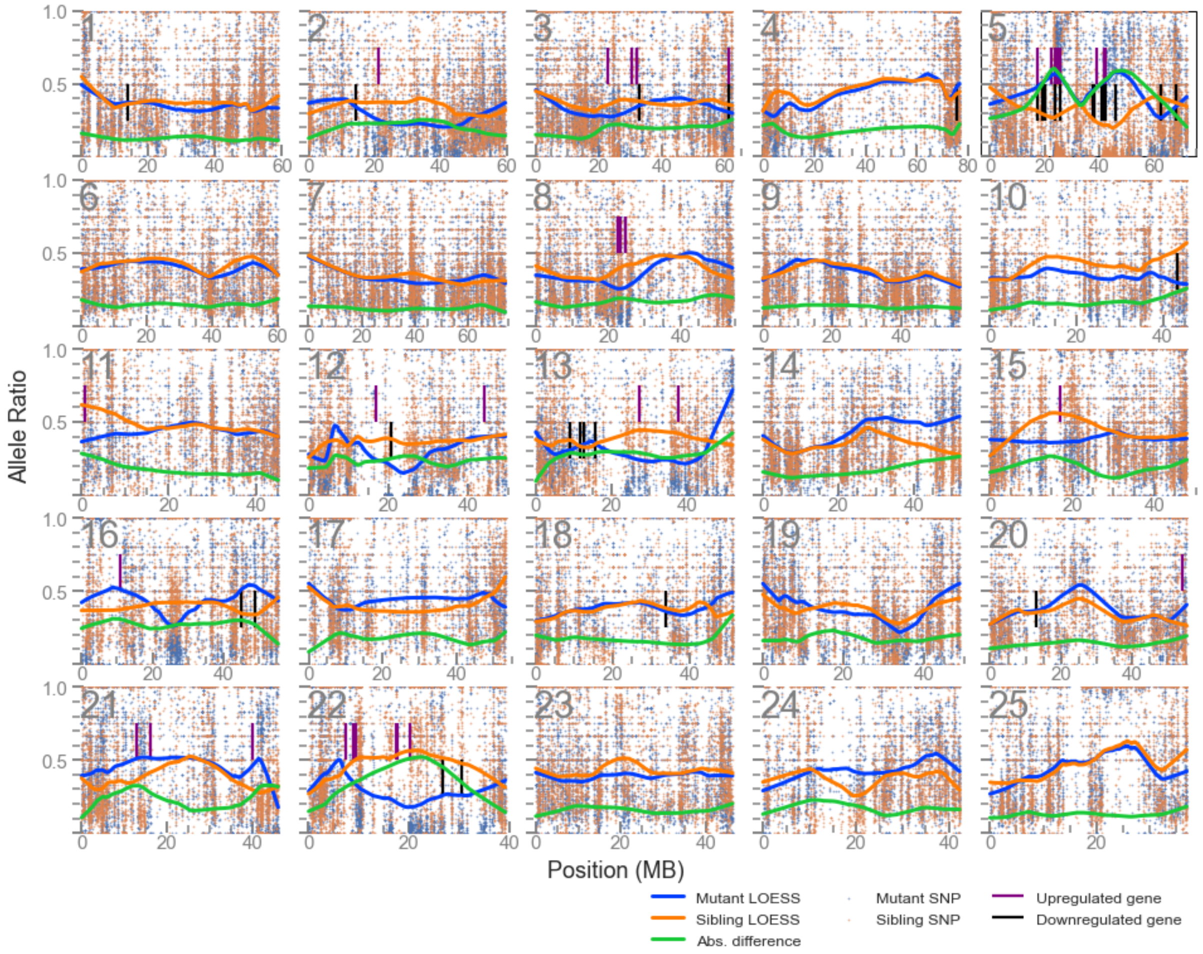

Allele u505 (CRISPR mutant), gene: dmist (chr5, 19.9Mbp)

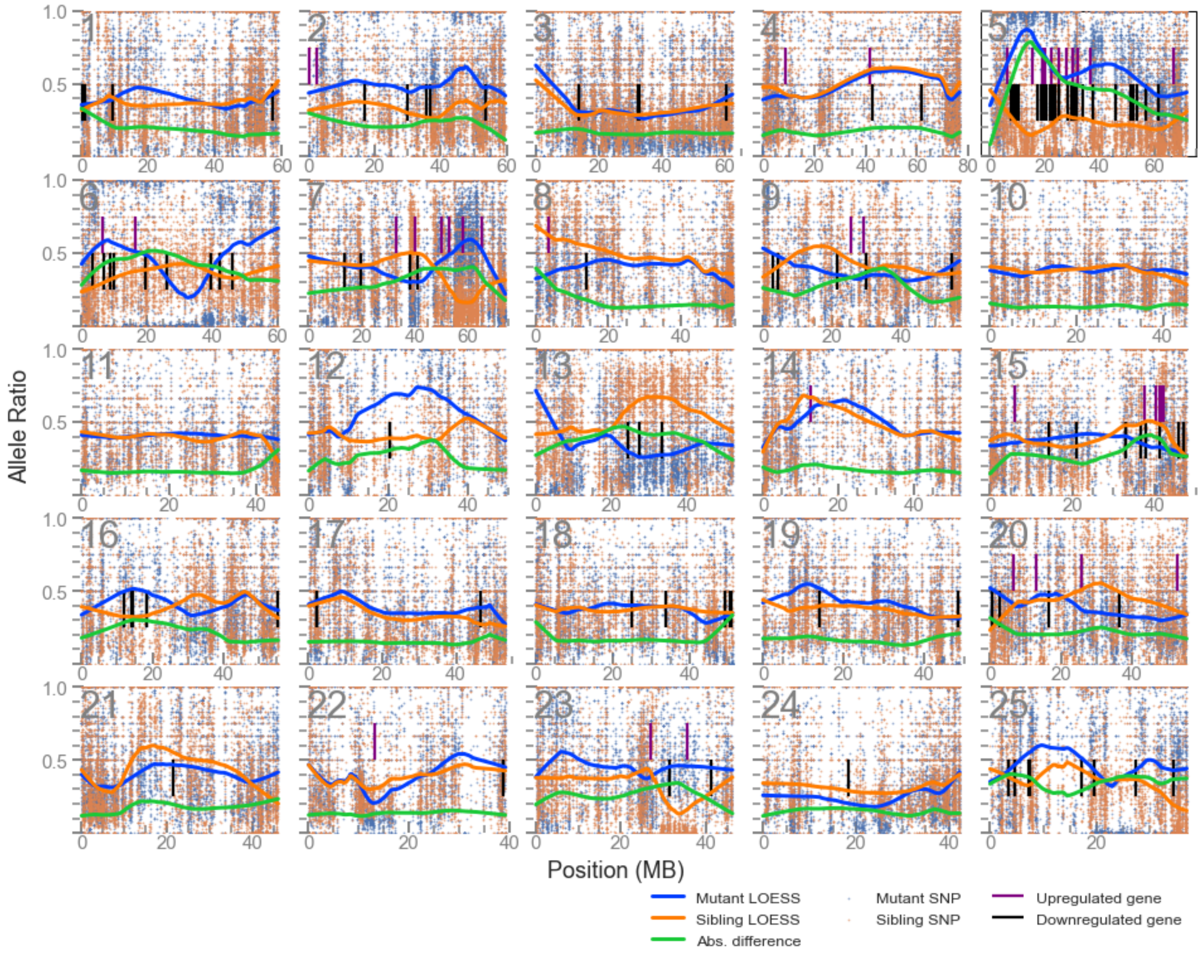

Allele la015577 (viral insertion), gene: dmist (chr5, 19.9Mbp)

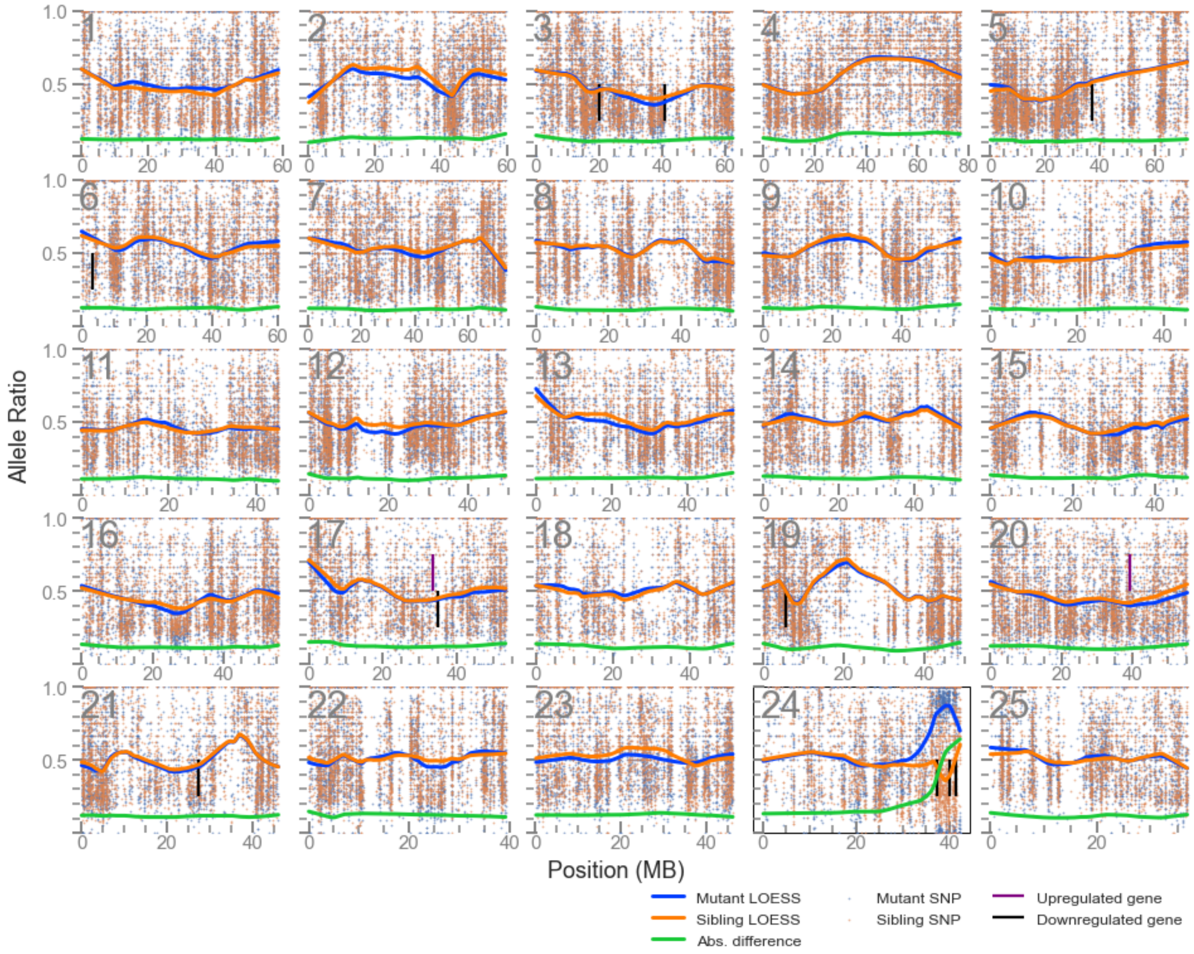

Allele nl14 (308), gene: lama1 (chr24, 41.6Mbp)

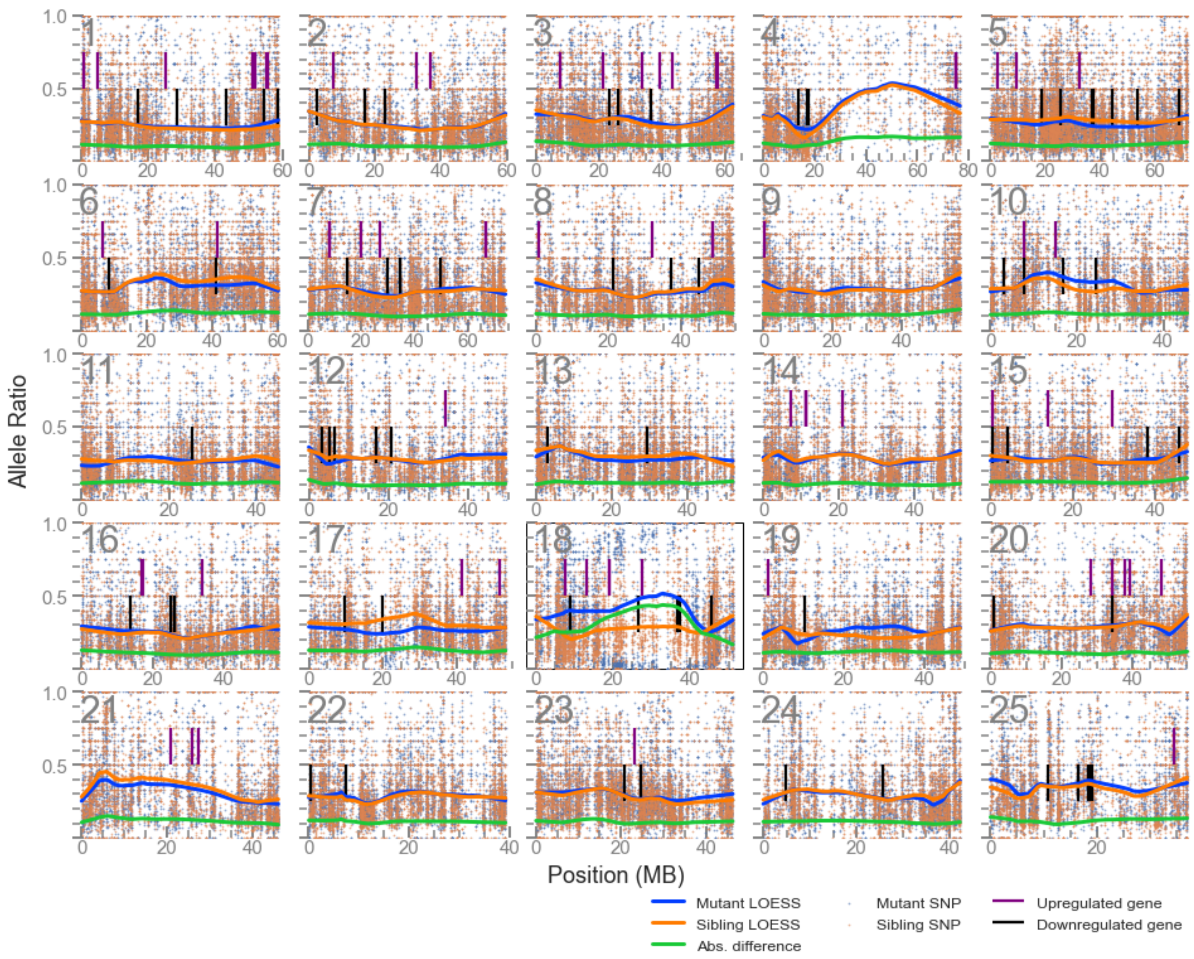

Allele nl13, gene: yap1 (chr18, 37.2Mbp)
